# Comparison of Training Strategies for Autoencoder-Based Monochromatic Image Denoising

**DOI:** 10.3390/s23125538

**Published:** 2023-06-13

**Authors:** Piotr Jóźwik-Wabik, Krzysztof Bernacki, Adam Popowicz

**Affiliations:** Faculty of Automatic Control, Electronics and Computer Science, Silesian University of Technology, Akademicka 16, 44-100 Gliwice, Poland; piotr.jozwik-wabik@polsl.pl (P.J.-W.); kbernacki@polsl.pl (K.B.)

**Keywords:** image denoising, Gaussian noise, autoencoder

## Abstract

Monochromatic images are used mainly in cases where the intensity of the received signal is examined. The identification of the observed objects as well as the estimation of intensity emitted by them depends largely on the precision of light measurement in image pixels. Unfortunately, this type of imaging is often affected by noise, which significantly degrades the quality of the results. In order to reduce it, numerous deterministic algorithms are used, with Non-Local-Means and Block-Matching-3D being the most widespread and treated as the reference point of the current state-of-the-art. Our article focuses on the utilization of machine learning (ML) for the denoising of monochromatic images in multiple data availability scenarios, including those with no access to noise-free data. For this purpose, a simple autoencoder architecture was chosen and checked for various training approaches on two large and widely used image datasets: MNIST and CIFAR-10. The results show that the method of training as well as architecture and the similarity of images within the image dataset significantly affect the ML-based denoising. However, even without access to any clear data, the performance of such algorithms is frequently well above the current state-of-the-art; therefore, they should be considered for monochromatic image denoising.

## 1. Introduction

Monochromatic images are widely used in low-level computer vision scenarios where the most important information is the intensity of the received signal. The most common examples of practical applications may be magnetic resonance imaging (MRI), computed tomography (CT), or astronomical observations, where the resulting images contain information that is usually displayed in grayscale. Unfortunately, in many situations, these images are obtained with additional noise, which significantly degrades their final quality. This can further complicate data analysis and even lead to incorrect conclusions. A good example would be a medical diagnosis, which, if incorrectly made, can result in numerous complications and even pose a threat to patients.

To combat this, numerous noise reduction solutions have been proposed over the years. The largest group of denoising methods is deterministic algorithms operating on a single noisy image. This group can be further divided into two separate types: local and non-local algorithms. The first one operates only on a small patch of the image, not on the whole image, hence the term “local”. It consists of, among others, simple filters that use information on mean and variance values [1], weighted median filters [2], Wiener filters [3], and bilateral filters [4]. Unfortunately, these techniques do not perform well when the noise level is high because the correlations of neighboring pixels are too seriously disturbed.

For this reason, the second type of algorithm has been developed, non-local methods, which take information from the entire processed image. The pioneering work in this field was the non-local mean filter (NLM) [5], which proved to be superior to local-based methods. This algorithm has been further improved in the works [6,7,8], leading to the most advanced method, BM3D (block-matching and 3D filtering) [9], which is currently the state-of-the-art deterministic algorithm for noise reduction.

Of course, deterministic algorithms are not the only approaches to image denoising. Of the other techniques, we can mention K-SVD (Singular Value Decomposition) [10], adaptive principal components [11], wavelet domain Gaussian scale mixture [12], probabilistic Gaussian Mixture Model [13], and Bayesian approaches [14,15]. However, at this point, it should be noted that some of them turned out to be worse than BM3D in terms of real image processing [16]. Therefore, BM3D can be treated as a reference point in the further analysis of the performance of noise reduction algorithms. Moreover, if any other deterministic algorithm is published, it is always compared against BM3D. Using such a filtering technique as a comparison is highly recommended in any work on image denoising.

Apart from the techniques mentioned, attempts have been made to develop denoising tools using neural networks, among which the works [17,18,19,20] should be mentioned. In recent years, machine learning approaches have surpassed the noise reduction capabilities of other algorithms in numerous areas. However, many of these solutions find limited application due to their disadvantages in preparation for work. Neural networks are complex mathematical models that are prepared for operation through a process called training. It consists of processing noisy images that are then compared with noise-free images. The problem is that in many applications, access to noise-free images is severely limited, and in some cases we may not even have access to them.

To address this issue, steps have been taken to bypass this requirement, leading to a number of new network training techniques: Noisy-As-Clean [21], Noisier2Noise [22], Noise2Void [23] with its probabilistic enhancement [24], Self2Self [25], Noise2Self [26], and Noise2Noise [27]. The names of these methods describe how they deal with the lack of a clean input image and what the training scheme looks like.

The main goal of our research is to determine the ability of neural networks to denoise images having only access to a large amount of noisy images without any clear image. If these approaches turn out to be effective, it will allow them to be used on a wider scale in situations in which we deal only with noisy data (e.g., in astronomical observations, where the reference is never accessible). For this reason, we validated the noise-to-noise training approach and compared the results with those obtained during training on images partially affected by noise or even noise-free images.

For testing, we chose a simple convolutional autoencoder architecture, which is characterized by additional compression abilities. We decided on this for two reasons. First, we concluded that a simple network should be chosen so that the results would depend mainly on the learning approach and not on the complexity of the network. Second, we were interested in the ability to control the compression quality, which we intended to test as part of noise reduction. Furthermore, we checked how the complexity of such a network wouldw affect the final results for a variety of learning approaches.

The tests were carried out on the widely used MNIST and CIFAR10 datasets of images (MNIST stands for Modified National Institute of Standards and Technology database while CIFAR for Canadian Institute For Advanced Research). These extended collections are widely accepted in the image processing community. The sets are very good examples of correlated (MNIST) and uncorrelated (CIFAR) collections of data. Three different, arbitrarily selected, levels of Gaussian noise were employed in our study. All results were assessed in comparison with the outcomes of the state-of-the-art deterministic algorithms as well as with the unprocessed noisy data. For this comparison, a widely used measure of Peak Signal-to-Noise Ratio (PSNR) was used.

Some limitations of our experiment should be also noted. The first limitation is the small resolution of analyzed images (32 × 32 pixels for both datasets). For large images, one needs larger architectures of the autoencoder, which require more powerful GPU units. The selected dataset, on the other hand, makes these suitable for research using relatively small architectures and with a limited access to large computing resources.

Another limitation is the type of noise utilized. Gaussian noise is indeed the most frequently found in the monochromatic images, but other kinds can be sometimes found (such as impulsive or uniform). The mixture of the noise types can be also visible in some real data.

Finally, the utilized PSNR comparison coefficient, although it is the most widespread quality indicator, has some limitations. Other quality metrics have been developed, such as SSIM (Mean Structural Similarity Index) [28]. However, they do not expose the photometric differences, rather concentrating on the imitation of image assessment by the human eye.

## 2. Materials and Methods

### 2.1. Learning Approaches

In a practical application, the network is supposed to operate in such a way that it receives a noisy image as input and processes it so that a noise-free image should appear at the output. This does not mean, however, that the network can be prepared to work only according to this scheme. Given access to different types of data, we can train the network in different ways.

The experiment included three available data types. The first includes images containing noise; we refer to this type of data as **Noisy**. The next one contains completely noise-free images, which are not available in most cases; this type is called **Reference**. The last one has images with a somewhat reduced level of noise, **Cleared**. The **Cleared** data may be obtained by preprocessing the noisy images, such as by applying filtering, calculating an averaged image (as is done in the stacking of astronomical images), or may simply represent images obtained under improved conditions (less noisy image sensor, more light, etc.).

Based on these data types, six different learning techniques can be distinguished, which are presented in Table 1 (abbreviations included). Importantly, the names of these methods do not describe what the network will output but rather what its output is compared to in the learning process.

These learning modes can be divided into three fundamentally different groups that allow us to check how the network will perform under any operating conditions. The first group can be considered modes 1 and 2—during learning, the network deals with images completely devoid of noise, thanks to which it can accurately assess what to change in its structure to obtain the expected **Reference** noise-free image. A network trained in this way is expected to show the highest filtering quality. Since in most practical applications it is not possible to acquire completely noise-free images, these modes should be treated rather as theoretical.

The second group consists of modes 3 and 4, in which the images already denoised to some extent are used. We expect them to perform reasonably well, and we want to check their efficiency in comparison to theoretical modes 1 and 2.

The last group includes modes 5 and 6, which describe the most common case where only **Noisy** images are available. Two cases are considered here: in 5, we examine how the network learns when it has to output an image with the same noise realization, while in 6, we check what happens when the network has to reproduce an image contaminated with different noise realizations. It seems to be very interesting to check whether filtering capabilities appear in networks trained to output the same or another **Noisy** image.

### 2.2. Application of Neural Networks

**Figure 1:**
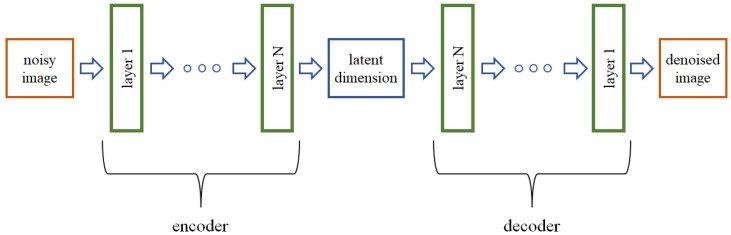
Structure of the autoencoder neural network.

For the purposes of the experiment, a structure of the autoencoder (AE) neural network has to be selected. It is a structure composed of two subnetworks, an encoder and decoder, built of layers arranged symmetrically in relation to the latent dimension of a one-dimensional central array (see Figure 1). The network works in such a way that it takes an image of a certain size (in our case, 32 × 32 pixels) as input; then, the encoder converts it to a latent dimension, and finally the decoder converts it to an output image of the same size as the input image. The size of the latent dimension (later referred to as l_dim) is multiple times smaller than the size of an input image, owing to which AEs find numerous applications in data compression.

We found this compression ability of AE very useful for noise reduction. We concluded that by fine-tuning the l_dim parameter, we could fine-tune the degree of compression and make it a lossy one. Moreover, in many cases, noise can be treated as the most complex information and thus the most likely to be lost. For this reason, we also evaluated the influence of the length of the latent dimension on the denoising of the input images.

Each of the mentioned AE layers consists of three separate elements, a 2D convolution layer, batch normalization layer, and hyperbolic tangent activation function, among which the most important is the number of channels (feature maps) in individual convolution layers. This number, as well as the number of layers and their arrangement in relation to each other, is the second most important parameter of the network, significantly affecting the obtained results. That is why we grouped these parameters into different sets, which we call separate architectures, and then checked their impact on the final result in the experiments carried out.

Many different loss functions are currently used in the neural network training process, but the most popular are the mean absolute error (MAE or $L_{1}$) and the mean square error (MSE or $L_{2}$). In our experiment, we decided to use both of these loss functions, and in addition, we also used the sliced Wasserstein distance ($L_{SW}$) [29]—a less popular metric, which has, however, found some applications in teaching neural networks [30,31], also in the case of autoencoders [32]. Therefore, the difference *f* between the network output image $im_{out}$ and the expected image $im_{exp}$ is calculated as in Equation (1).

(1) $$f\left(im_{out},im_{exp}\right)=L_{1}\left(im_{out},im_{exp}\right)+L_{2}\left(im_{out},im_{exp}\right)+L_{SW}\left(im_{out},im_{exp}\right)$$

### 2.3. Evaluation of the Experimental Results

In order to numerically compare the quality of the processed images, we decided to use PSNR [33], which is probably the most popular quality indicator used to assess the efficiency of image filtering, compression, etc. Its value is calculated based on the mean squared error between the compared images, as well as the maximum possible pixel value of the image. The higher the PSNR value (expressed in decibels), the greater the similarity between the examined images; for identical images, the PSNR equals infinity. In our case, the test datasets consist of 8 bit images. For this bit depth, images with PSNR values of 30 dB and above can be considered high quality.

For each image from the test set, we calculated the PSNR values. In this way, we obtained a benchmark set against which we could compare the noise reduction by the methods used. In addition to the neural network approaches used, we also used BM3D and NLM to test our solutions against the state-of-the-art denoising methods. This allowed us to assess in which cases the use of such methods can be useful and provide considerably better results.

### 2.4. Experiment Dataand Noise Modeling

As an experiment data set, the well-described and widely used MNIST dataset [34] was chosen. It is a collection of many thousands of handwritten digit images that, for implementation purposes, were scaled to a size of 32 × 32 pixels, and their values were normalized to [−1; 1]. These images, although quite similar, are characterized by a noticeable diversity that prevents the network from overtraining. Moreover, they are easy to assess visuallyand have a noticeable similarity to each other. Therefore, the use of MNIST allows us to evaluate the performance of the tested approaches in situations where we want to denoise a hermetic, specialized data set.

For research, the MNIST dataset was divided into three separate subsets. We found that the digits 4, 7, and 8 differ in shape from the others and extracted a test subset from them, which was solely used to evaluate the performance of the trained networks. The remaining images were divided with a 17:3 ratio for each type of data (corresponding digits) into a training and validation set.

In addition, we used the CIFAR-10 dataset [35], which also contains several thousand 32 × 32 pixel images. Originally, they were saved as color images, but we converted them to grayscale for the purposes of the experiment. This set is much more diverse, which allows us to determine the degree to which the application of the tested learning approaches depends on the complexity of the set. This set was divided in a similar way as MNIST. However, the selection was random and not based on image labels. The division of both datasets is described in Table 2.

**Figure 2:**
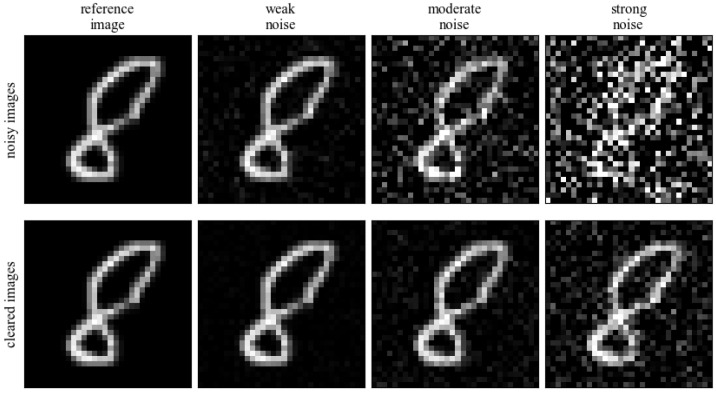
Simulated noise power levels on a sample image.

On such a divided dataset, we conducted research on the impact of noise on the quality of the processed data. Based on the visual assessment of image degeneration, we selected three levels of Gaussian noise: weak, medium, and strong. For the selected noise levels, the **Noisy** data were modeled using the sigma parameter (values of 0.1, 0.15 and 0.85 were chosen, respectively as weak, median, and strong noise). The **Cleared** images were also created as images with reduced noise level (values of 0.01, 0.015, and 0.085). This kind of images represents the less noisy examples, which can be trained in pairs with their **Noisy** versions. Importantly, during the training of the network, the new noise was inputted in each learning iteration in order to increase the size of the training set as much as possible. Examples of **Noisy** and **Cleared** images in relation to the original image are shown in Figure 2.

### 2.5. Experiment Summary

**Figure 3:**
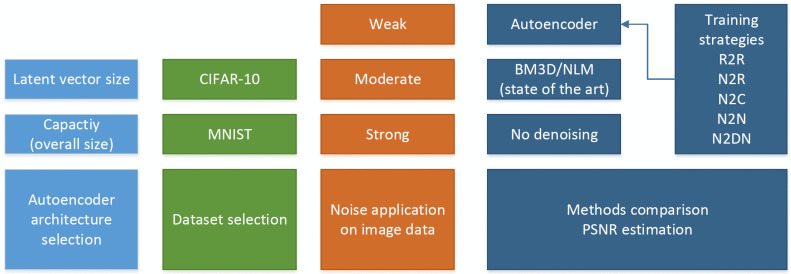
The flow chart of the performed experiment.

The flow chart of the experiment is given in Figure 3. We wanted to highlight the range and variety of included parameters and aspects (architectures, datasets, and noise levels). In the comparison of methods, we highlighted the crucial elements such as the various training approaches of the autoencoder, as well as a comparison with the current state of the art and with the images not treated by any denoising technique. The flow chart should be useful for a better understanding of the consecutive parts of our experiment.

## 3. Results

Network performance tests were carried out for five different architectures characterized by different numbers of channels (feature maps) in convolutional layers and thus a different number of all parameters. During the fine-tuning of the sizes of the used auto-encoders, we decided to test the smallest networks possible. This allowed us to fully assess the complexity of the problem and the noise reduction capabilities of each learning approach. Therefore, we made architectures 1 and 2 very small and architectures 3 and 4 slightly larger but still relatively small. Moreover, we used them to test how their performance would be affected when one small layer was added, that is, whether only the overall number of parameters is important or the number of individual layers as well. To compare their efficiency with a larger network, we implemented architecture 5, which is much larger than the others.

Furthermore, tests were carried out for four different sizes of latent dim: 8, 16, 32, and 64 (corresponding to the approximated compression ratios: 0.78%, 1.56%, 3.13%, 6.25%). The applied compression was considerable in all cases, which allowed for a full evaluation of its impact on the denoising process. The AEs used are presented in Table 3—the range of parameter numbers for each network results from the use of different latent dim sizes.

Due to the fact that the impact of individual algorithms was examined on the entire data sets, the results were presented in the form of boxplots. Boxplots are good at displaying the scatter of data around specific values. The edges of the box indicate the first and third quartiles, the crossbar indicates the average value of the set, and all the accepted resulting values are between the ends of the whiskers. The whiskers extend to points that lie within 1.5 IQRs (the interquartile range) of the lower and upper quartiles. All results that fall outside this range are outliers and rejected from the graph. In each of the graphs, the Y-axis describes the PSNR values in dB, and the X-axis divides the results into different latent dimension values, as well as a reference group that consists of three components: the results for the BM3D and NLM algorithms and, most importantly, the raw PSNR values for the **Noisy** test set.

**Figure 4:**
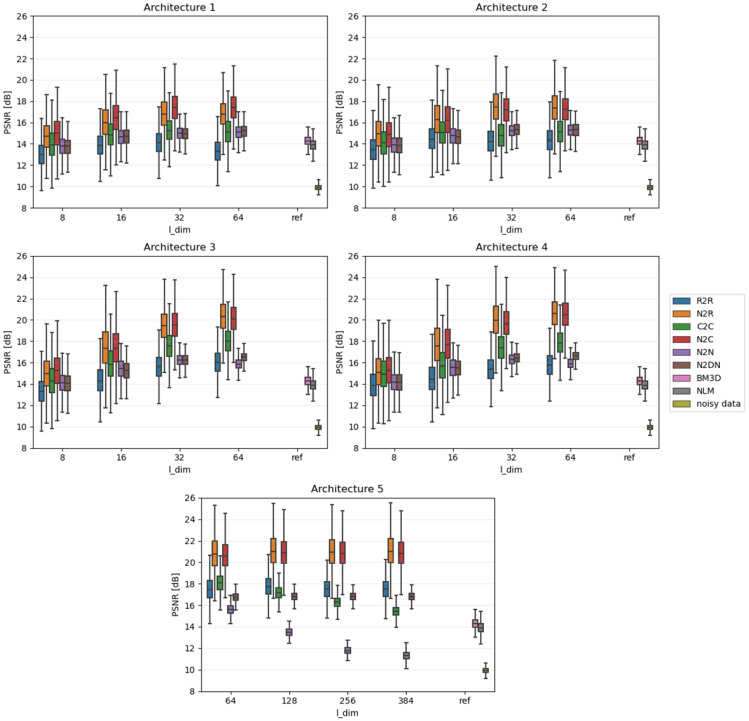
Results for strong noise power (MNIST).

**Figure 5:**
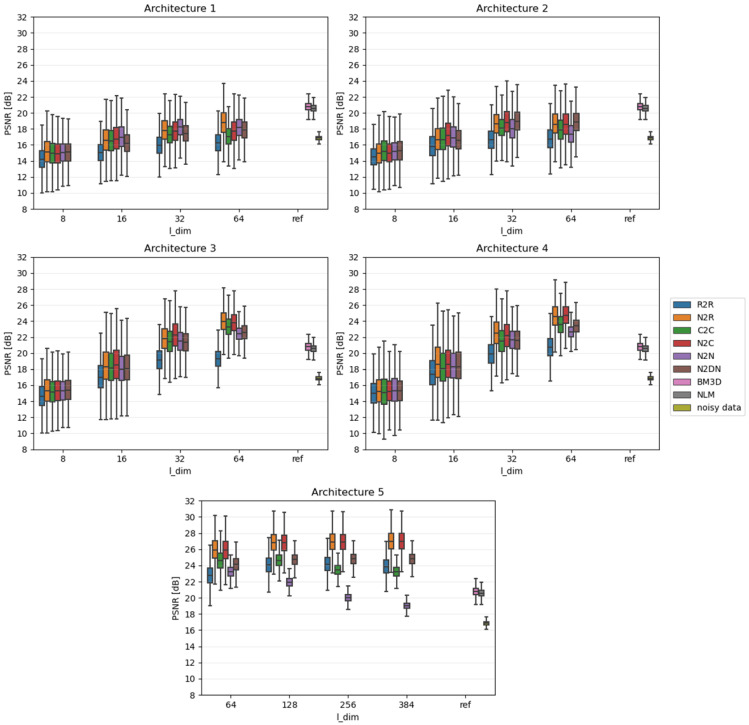
Results for moderate noise power (MNIST).

**Figure 6:**
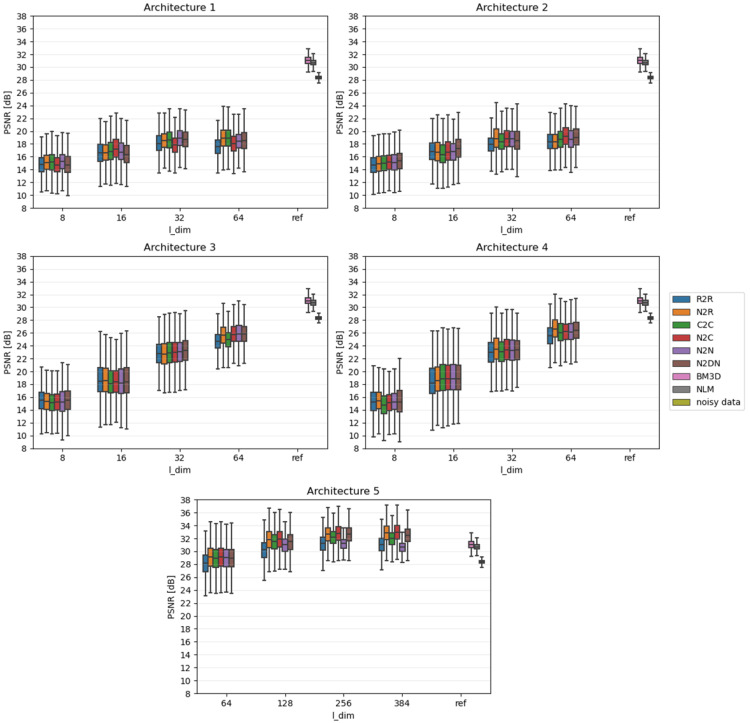
Results for weak noise power (MNIST).

**Figure 7:**
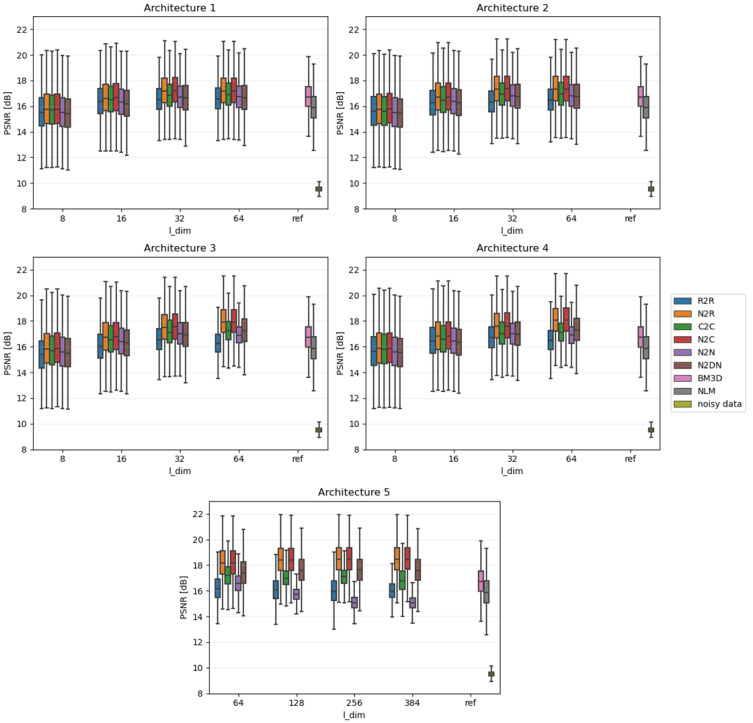
Results for strong noise power (CIFAR-10).

**Figure 8:**
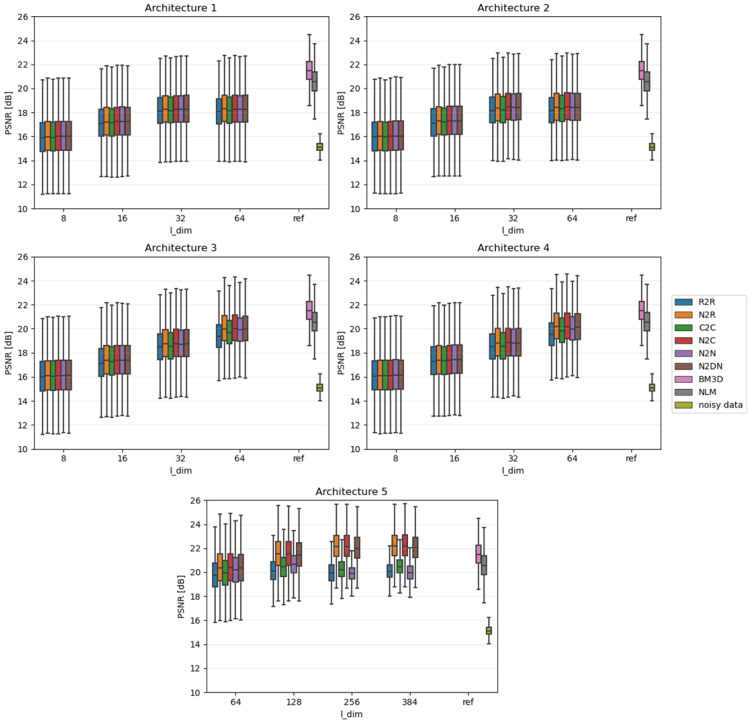
Results for moderate noise power (CIFAR-10).

**Figure 9:**
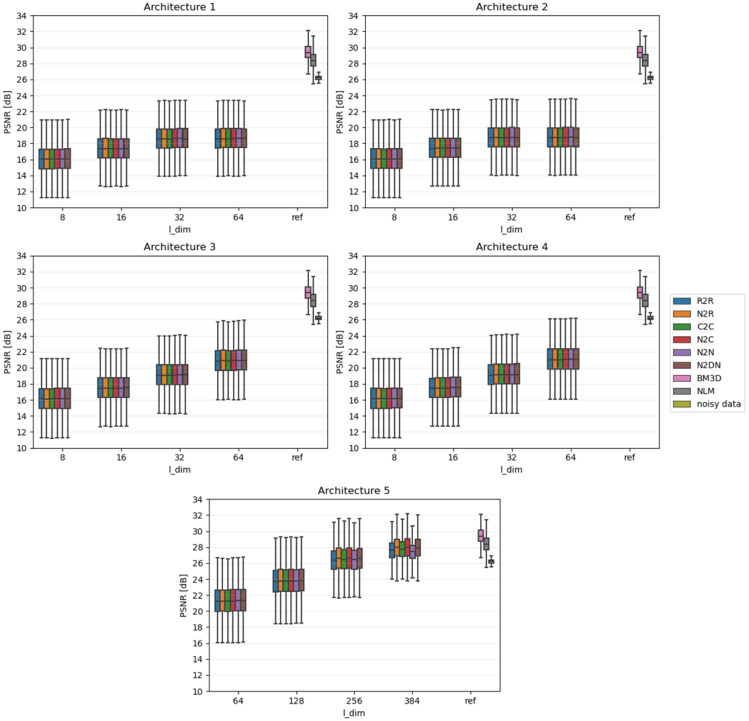
Results for weak noise power (CIFAR-10).

Figure 4, Figure 5 and Figure 6 show comparative plots for each of the tested noise powers for the MNIST dataset, and Figure 7, Figure 8 and Figure 9 for the CIFAR-10 dataset.. In addition to the results of the PSNR of denoised images, we show the PSNR of **Noisy** images as “noisy data” (see the last position in the legends). This presents the reference level of image degradation if no algorithm was applied.

**Figure 10:**
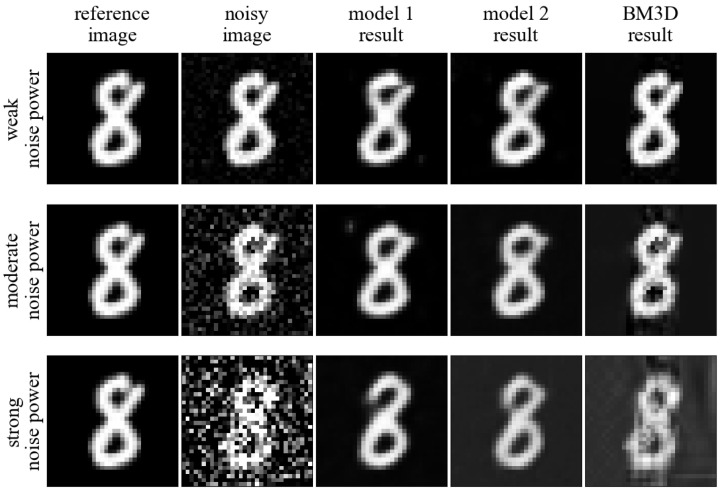
Visual comparison of different approaches performance.

In Figure 10, we show a visualization of the application of the algorithms tested on an example image. In each of the rows, the performance is presented for a different noise power (weak, moderate, strong). The first column shows clean (**Reference**) images, the second column shows images with added noise, the third and fourth columns show images denoised by the selected networks, and the fifth column shows images denoised by the chosen deterministic algorithm. As a deterministic algorithm, BM3D was chosen over NLM because it achieves better results. N2C (model 1) and N2DN (model 2) networks based on architecture 5 and the length of the latent dimension 128 (12.5% compression ratio) were selected as examples. This corresponds to the most interesting cases where only **Noisy** data were available during training, and attempts are made to process it through relatively small networks with a high degree of compression.

## 4. Discussions

The overall performance of applied learning approaches is largely dependent on the size of the networks used and the considered dataset. When dealing with tightly related data, such as that presented in MNIST, we can get noticeably different results than with a much less inter-correlated dataset, such as CIFAR-10. For this reason, the discussion can be divided into several parts. In the first two, we focus mainly on MNIST results for smaller (architectures 1–4) and larger (architecture 5) networks. Then, we discuss the differences between results for MNIST and CIFAR-10 datasets. The fourth part contains final remarks that focus on the usefulness of each learning approach. Finally, the last part describes the limitations of the experiment.

### 4.1. Smaller Networks (MNIST Dataset)

As expected, slightly larger networks (3 and 4) in most cases achieve better results than smaller networks (1 and 2). Moreover, adding an extra network layer causes some changes; however, they are small, and thus it is difficult to assess whether they simply result from the presence of an extra layer or the greater number of network parameters. On the other hand, the influence of the latent vector size (l_dim) is noticeable: with its increase, the noise reduction capabilities of the considered networks improve. This topic is covered in more detail in the latter subsection.

In the case of strong or moderate noise, small networks can achieve good results—sometimes better than applied state-of-the-art deterministic algorithms. Despite this, they stand out noticeably in the case of weak noise power; this is due to the fact that images only slightly affected by noise still show very high quality. Therefore, to achieve high PSNR, the network should not change the image too much. Unfortunately, this is not the case seen in small architectures trained to modify images to remove noise.

### 4.2. Larger Networks (MNIST Dataset)

For the case of minimal noise, a complex network is able to significantly improve the input image and even surpass deterministic algorithms in this respect. The impact of latent vector size changes on the results is again clearly visible—the more information such a network remembers, the less compression to which it will subject the key data, and the better it is able to reproduce them.

Considering the higher noise, the situation looks slightly different, as was already seen in small networks. Most of the approaches used are at some point independent of latent vector size, yet the N2N and C2C approaches are gradually starting to degrade in performance. This is because these methods force a network to repeat the input at its output, thus enhancing only the noise-memory skills of the network. Interestingly, the R2R method basically works the same; however, it shows a negligible decrease in efficiency.

### 4.3. CIFAR-10 Results

The variations between the different learning approaches are in most cases small—much smaller than in the case of the MNIST set. When the quality of denoising becomes close to that represented by the deterministic algorithms, the mentioned differences become more pronounced (i.e., for strong noise). This leads to conclusions analogous to the ones described in Section 4.1 and Section 4.2.

It should be emphasized that, unlike for MNIST, for CIFAR, it is very difficult for networks to outperform current state-of-the-art deterministic algorithms. This is mainly due to the much greater diversity of the dataset under study. The autoencoder could not learn the characteristics of expected images in such a case. This suggests that the best use for autoencoder-based denoising would be the datasets that are noticeably inter-correlated, as is true for MNIST dataset.

### 4.4. Final Remarks

Since the experiment on the CIFAR-10 dataset indicated clearly that the use of autoencoder-based denoising should be considered mainly for images that represent very similar characteristics, such as in the MNIST dataset, our final remarks, given below, are limited to this application.

In most cases, the N2R and N2C approaches achieve the best performance, with the difference between them usually being small. Next in efficiency are the results for R2R and N2DN, which are also able to significantly improve the quality of noised images. In practical applications, however, we usually do not deal with **Reference** images, and therefore we can only use N2C and N2ND on a large scale. However, this is not a problem, because when comparing N2C with N2R, we conclude that for the best network performance, we do not need a perfect **Reference** image, but only one cleaned image is enough. In the absence of this, we can use the N2DN approach and still expect good results, exceeding the achievements of state-of-the-art deterministic algorithms.

When dealing with images strongly affected by noise, even a small network is able to produce great results, but in the case of little noise, the problem is not the noise reduction itself but the preservation of the original parts of the image unchanged, which is associated with an increase in network complexity. That is why it is better to use a more complex network and then tune its performance by adjusting by changing the degree of data compression (the size of latent dim), which proved to be one of the most important autoencoder parameters. However, in the cases of larger networks, changing l_dim in the range of small compression ratios does not cause noticeable changes (except for N2N and C2C). Moreover, it can be seen that the N2R and N2C methods definitely dominate in terms of efficiency, especially when dealing with strong noise power; the stronger the noise, the more they stand out from other approaches. In other words, blindly increasing the network size does not always lead to better results.

In all tested cases, noise reduction using deterministic algorithms resulted in a noticeable improvement in the processed data. However, as the noise level increases, the BM3D and NLM’s denoising capabilities decrease. In the case of closely related data sets, we were able to train even small networks that were able to surpass them. Moreover, in many cases, the superiority of the autoencoder was seen regardless of the method of training. We conducted our research on a simple architecture, so it can be hypothesized that when using a much more complex network, the difference might be noticeably greater.

### 4.5. Experiment Limitations

The obtained results allow us to state that the autoencoder structure can be effectively used to improve data sets containing only **Noisy** images and surpass other denoising methods in this task. However, our goal was not to create or find the best possible novel network but to evaluate learning techniques on easy-to-train and basic architectures, for various scenarios of available data (i.e., noisy, cleared, reference). Therefore, it should be borne in mind that if other networks structures are used, the results might still differ from these obtained in our experiment.

Moreover, we used solely monochromatic images for the study, and the noise was modeled only as Gaussian. In many real cases, the noise can be more complex and not uniformly distributed in the image. Attention should also be paid to larger real life images, which may contain a much greater degree of detail.

## 5. Conclusions

The capabilities of deep learning techniques and neural networks architectures are expanding every day. One of the important applications of these solutions is the denoising of monochrome images. Although the fact is that the capabilities of such algorithms often surpass current state-of-the-art deterministic methods, the techniques for teaching ML solutions can lead to significantly different efficiencies.

In this paper, we focus on a range of learning methods given different training sets of images. We examine how methods that use real-world data perform relative to learning techniques that require access to ideal, noise-free data.

Our results show that lack of access to benchmark data is not a big problem for teaching networks. For example, models trained for the task of converting one **Noisy** image to another **Noisy** one (i.e., the **Noisy** to **Noisy** method) are able to present capabilities similar to the models learned on the best possible data. Moreover, such models usually outperform state-of-the-art methods, suggesting the strong need to consider them when building noise reduction solutions. Nevertheless, we have observed that the benefit of having ideal images is usually apparent, although sometimes small. Access to data with reduced noise (i.e., data that are better than the input but not ideal and noise-free) should always be utilized in the learning process.

Further research work will be carried out in order to understand the impact of network learning techniques on the efficiency of improving not only the noise aspects, but also the resolution of images.

## Figures and Tables

**Table 1:** Different learning approaches.

Name	Autoencoder Input	Expected Output
Reference to Reference (**R2R**)	A reference image	The same reference image
Noisy to Reference (**N2R**)	A noisy image	A reference image
Cleared to Cleared (**C2C**)	A cleared image	The same cleared image
Noise to Cleared (**N2C**)	A noisy image	A cleared image
Noisy to Noisy (**N2N**)	A noisy image	The same noisy image
Noisy to Different Noisy (**N2DN**)	A noisy image	A different noisy image

**Table 2:** Data sets division into subsets.

Dataset	Traning Set	Validation Set	Test Set
MNIST	41,700	7358	20,942
CIFAR10	36,000	6000	18,000

**Table 3:** Details of utilized architectures.

Architecture	Number of Channels	Approx. Number of Parameters
1	[4, 8, 4, 8]	2500–6100
2	[4, 4, 8, 4, 8]	2800–6400
3	[8, 12, 16, 20]	12,750–21,750
4	[8, 8, 12, 16, 20]	13,950–22,950
5	[48, 72, 96, 120]	404,700–458,500

## Data Availability

Data available from the authors upon request.
